# Cholera and Inoculation

**Published:** 1888-09

**Authors:** 


### CHOLERA AND INOCULATION.

A cable message from Paris, to the secular press of this country, recently announced that "The scientific event of the week, of course, has been the Russian Dr. Gamaleia's alleged discovery of a method of anticholera inoculation," and the reported statement by Pasteur that "I am so thoroughly convinced of Gamaleia's success that I would to-morrow, were there need, allow a test to be made upon my own person, and in so doing should run small risk." After the notoriety obtained in Spain by Ferran, and the outcome of his method of inoculation, the medical profession and the public have been slow to accept assurances of security from cholera, or even from septicæmia, as a result of inoculation, but the confident statement, alleged to have been made by so good authority as Pasteur, and the method of procedure of Dr. Gamaleia, may give some warrant of hope that prophylaxis may yet be secured in some such way against that scourge.

Pasteur is quoted as having said further: "Of course there was nothing novel in the idea of inoculation against cholera. The difficulty lay in applying the well-understood principles of vaccination to this new case as I had already applied them to hydrophobia, and as some one will one day apply them to tuberculosis and other diseases." All will wish that the confident anticipations of this eminent scientist and experimenter may be realized and that he may live to witness the fact. In the mean time, for the sake of humanity and science, let us hope that the claims of Dr. Gamaleia for his discovery will not share the fate of those made by the once notorious Ferran.

